# Viperin interaction with mitochondrial antiviral signaling protein (MAVS) limits viperin-mediated inhibition of the interferon response in macrophages

**DOI:** 10.1371/journal.pone.0172236

**Published:** 2017-02-16

**Authors:** Jia Shee Hee, Peter Cresswell

**Affiliations:** Department of Immunobiology, Yale University School of Medicine, New Haven, Connecticut, United States of America; University of Tennessee Health Science Center, UNITED STATES

## Abstract

Viperin is an antiviral protein that is upregulated by interferons and by ligands for a variety of innate immune receptors. It possesses diverse capabilities and functions in an array of viral infections. Studies have shown that it appears to be particularly important in defence against RNA viruses, such as West Nile, Dengue, and Chikungunya viruses, although the specific mechanisms involved are not well understood at the molecular level. Here we identify the mitochondrial antiviral signalling protein MAVS as a novel viperin interaction partner, most likely in mitochondria associated membranes, and characterize a more central, overarching role of viperin as a negative regulator of the interferon response, an ability that can be regulated by the viperin-MAVS interaction. This suggests a novel mechanism of viperin action in immune defence against RNA viruses by which it may prevent pathology from excessive immune responses.

## Introduction

Virus Inhibitory Protein, Endoplasmic Reticulum-associated, interferon-inducible (viperin) is the product of the interferon stimulated gene (ISG) *rsad2*. It has been reported to play an antiviral role against a number of viruses, including Human Immunodeficiency Virus [1], Hepatitis C Virus (HCV) [2, 3], West Nile Virus (WNV) [4, 5], Dengue Virus [4, 6], Chikungunya Virus [7], Influenza A Virus [8, 9], and Human Cytomegalovirus (HCMV) [10]. The postulated mechanisms of action vary widely, from the inhibition of viral budding by affecting lipid rafts in the case of influenza A to preventing viral replication by interacting with viral proteins in the case of HCV (reviewed in [11], [12]). Viperin has also been shown to participate in immune system cell signaling, modulating NFκB and AP-1 signaling in T cells [13] and the TLR-7 and TLR-9 pathways in plasmacytoid dendritic cells [14, 15]. To date, no unifying theory ties all the reported functional aspects together and our understanding of viperin is clearly incomplete. Mice lacking viperin appear normal and when infected by most viruses do not show a clearly distinguishable phenotype, perhaps because of redundancy in interferon-induced effector mechanisms. However, they are more susceptible to WNV [5] and Chikungunya virus infection [7], and WNV replicates better in macrophages lacking viperin [5]. WNV and Chikungunya virus are both positive strand RNA viruses, suggesting that viperin may have a particularly important role in such infections.

Mitochondrial anti*v*iral signaling protein (MAVS), an adaptor molecule downstream of the cytosolic RNA receptors retinoic acid-inducible gene I (RIG-I) and melanoma differentiation-associated protein 5 (MDA-5), is critical for the innate immune response to RNA viruses. After being activated by their RNA ligands, RIG-I and MDA-5 bind to MAVS, which then oligomerizes and recruits downstream signaling molecules, including STING, TRAF2, TRAF3, and TRAF6. This results in the activation and translocation of the transcription factors IRF-3, IRF-7 and NF-κB to the nucleus and leads to the production of IFNs and inflammatory cytokines [16, 17]. Given the key role of MAVS in defense against RNA viruses, we hypothesized that it might work together with viperin to regulate the antiviral response. Consistent with this idea, the HCMV protein vMIA inhibits MAVS-linked IFN induction by inhibiting downstream signaling [18, 19] and it also interacts with viperin, resulting in its translocation to mitochondria. Mitochondrial viperin induces a block in fatty acid β-oxidation combined with an increase in fatty acid biosynthesis, a process that plays a proviral role in HCMV infections by facilitating membrane formation [10].

Pathology in viral infections is often a result of excessive inflammation, and various mechanisms exist to regulate this. A splice variant of MAVS, known as miniMAVS, inhibits MAVS signaling and its downstream effects [20, 21]. The spectrum of ISGs, besides including antiviral effectors and positive regulators, also includes negative regulators such as SOCS and USP18 [22]. Multiple other regulators, such as LGP2, NLRX1, and PLK1, inhibit MDA-5-, RIG-I- or MAVS-dependent signaling [23–27]. One therefore cannot discount the possibility that viperin, in addition to being an antiviral effector, might also be a regulator of the interferon response.

Although MAVS was so named because it is found on mitochondria, it has also been shown to localise to peroxisomes and mitochondria-associated ER membranes (MAM), particularly upon the activation of its signaling pathways [28–30]. In initial experiments we determined that a fraction of viperin was also localized in MAM. We thus set out to elucidate the role of viperin in the MAVS-dependent signalling pathway and its potential role in defence against RNA viruses.

## Materials and methods

### Mice

C57BL/6 mice were purchased from Jackson Labs and maintained alongside viperin knockout mice [10]. MAVS knockout mice were a kind gift from the laboratory of Dr. Akiko Iwasaki. All mice were euthanized with carbon dioxide followed by spinal dislocation as per protocol designated by the Yale Animal Resources Center (YARC) and the Institutional Animal Care and Use Committee (IACUC), which approved this study.

### Plasmids and primers

pcDNA3.1-HADHA, pcDNA3.1-HADHB, pcDNA3.1-myc-his and pcDNA3.1-viperin have been previously described [10, 11]. All primers for making mutants were synthesized by the Keck facility at Yale University. Deletion and point mutants were made from pcDNA3.1-viperin via PCR using Turbo-Pfu (NEB). PCR products were digested with DpnI (NEB) for 5h at 37°C before being transformed into DH5α or Top10 cells (Thermo Fisher Scientific). Viperin was cloned into pRetroX (Clontech) by PCR using Taq-HiFi (NEB) and digested with restriction enzymes (NEB), ligated using T4 DNA ligase (NEB) into the digested vector backbone, and transformed into Stbl3 cells (Thermo Fisher Scientific). pCMV-FLAG-MAVS was a kind gift from the laboratory of Dr. Akiko Iwasaki. pCMV-SPORT6-ΔtmMAVS was a kind gift from the laboratory of Dr. Yorgo Mordis. pcDNA3.1-MDA5 and pcDNA3.1-RIGI were a kind gift from Dr. Shu Zhu in the laboratory of Dr. Richard Flavell. Plasmids were purified using Qiagen miniprep kits or kits from Origene and Zymo Research.

### Western blotting

Cells were harvested, washed with PBS, and lysed on ice for 30 minutes in lysis buffer (1% CHAPS (Pierce) in PBS containing a protease inhibitor cocktail tablet (Roche)). After centrifugation at 800g for 10 minutes at 4°C to remove debris, lysates were sparated by SDS-PAGE and transferred onto PVDF membranes (Millipore). The membranes were probed using antibodies against viperin (MaP.VIP) [8], or commercial antibodies to MAVS (Abcam and Santa Cruz), MDA-5 (Cell Signaling Technology), RIG-I (Cell Signaling Technology), calnexin (Enzo), Grp94 (Enzo), FACL4 (Abcam), TFPβ (LifeSpan BioSciences), Tim23 (BD Biosciences), and non-phosphorylated IRF3 and phosphorylated IRF3 (Cell Signaling Technology). Secondary antibodies conjugated to Horse Radish Peroxidase were purchased from Jackson ImmunoResearch. Blots were developed using SuperSignal West Pico Chemiluminescent Substrate (Thermo Fisher Scientific).

### Immunoprecipitation

To conjugate antibodies to beads, Protein G Sepharose beads (GE Healthcare) were rotated with anti-viperin antibody or anti-MAVS antibody (Santa Cruz) overnight at 4°C. Beads were washed once with PBS and twice with 0.2M borate buffer, pH 9.0. Dimethyl pimelimidate dihydrochloride (Sigma) was dissolved in the borate buffer to a final concentration of 5.2 mg/ml and added to beads, followed by rotation at room temperature for 30 minutes. Beads were washed twice with 1.2% ethanolamine-HCl buffer, pH 8.0, and rotated for 2 hours, washed twice with 100mM glycine, pH 3.0, and twice with PBS. Beads were added to CHAPS lysates of the cells and rotated at 4°C for 2 hours. After 4 washes with 0.1% CHAPS in PBS the samples were separated by SDS-PAGE and transferred onto PVDF membranes.

### Subcellular fractionation

RAW 264.7 cells were stimulated with universal type I IFN (PBL Interferon Source) in cell culture medium (DMEM, 10% BCS, 1% Pen/Strep) for varying lengths of time. Fractionation was performed using Percoll gradients as described [31].

### Confocal immunofluorescence microscopy

Cells were stained with MitoTracker Red (Thermo Fisher Scientific) for 30 minutes at 37°C, washed with PBS, and fixed with 2% paraformaldehyde in PBS for 15 minutes at room temperature. After washing in 10mM glycine in PBS and blocking with 5% normal goat serum (Invitrogen) in PBS, the cells were permeabilized with 0.5% saponin (Sigma) in 5% normal goat serum in PBS for 1 hour at room temperature. Staining was performed in permeabilization buffer. Primary antibodies used were the same as used for western blotting, except that a rabbit antiserum against calnexin was used. Secondary antibodies were the Alexa Fluor series from Thermo Fisher Scientific. Slides were mounted with ProLong Gold Anti-Fade Reagent (Thermo Fisher Scientific), viewed using a Leica SP8 model confocal microscope and the images were analysed using ImageJ software.

### Mouse bone marrow macrophage isolation and stimulation

Mouse bone marrow cells were isolated by flushing hind leg bones. Cells were counted and plated in culture medium (DMEM, 5% FBS, 1% Pen/Strep) containing 30% medium from L929 cells secreting macrophage colony stimulating factor (MCSF). After 6 days, resulting BMM were counted and re-plated at desired densities in culture medium with 5% MCSF-containing L929 medium. Experiments were performed the next day. IFN was added directly, while poly(I:C) (Sigma) and 5’ppp-dsRNA (Invivogen) were transfected into cells using Lipofectamine 2000 (Thermo Fisher Scientific) in Opti-MEM (Gibco). After 1 hour the cells were re-suspended in culture medium. Fluorescein-tagged poly(I:C) was purchased from Invivogen. All other ligands were a kind gift from the laboratory of Dr. Ruslan Medzhitov.

### Quantitative PCR

RNA was extracted from cells using the Qiagen RNeasy Mini Kit. This was converted to cDNA using the AffinityScript multi temperature cDNA synthesis kit (Agilent Technologies) and a BioRad PCR machine. Quantitative PCR was performed using SYBR Green from Life Technologies and Biotool. Analysis was performed using an Mx3000P (Stratagene) and the MxPro software (Agilent Technologies).

### Flow cytometry

Cells were harvested, washed with PBS, and collected using an Accuri C6 CSampler (BD Biosciences). Analysis was performed using FlowJo software. To test for activated caspase-3, cells were stained using the FITC Active Caspase-3 Apoptosis Kit (BD Pharmingen). To test for viperin, MaP.VIP was directly conjugated to Alexa 647 (Thermo Fisher Scientific).

### Luciferase reporter assay

293T cells were transfected with pISRE-firefly luciferase and various combinations of viperin or MAVS constructs as required. Each transfection had an equal amount of DNA, attained by adding pcDNA3.1-myc-his as a control where necessary. Cells were transfected with poly(I:C) if necessary. 25 to 30 hours after transfection, cells were washed with PBS and lysed in passive lysis buffer (Promega) for 15 minutes at room temperature with gentle shaking. Lysates were centrifuged to pellet debris and supernatants used in luciferase assays (Promega) read using MikroWin 2010 software.

## Results

### Viperin and MAVS localize to MAM independently of one another

**Fig 1:**
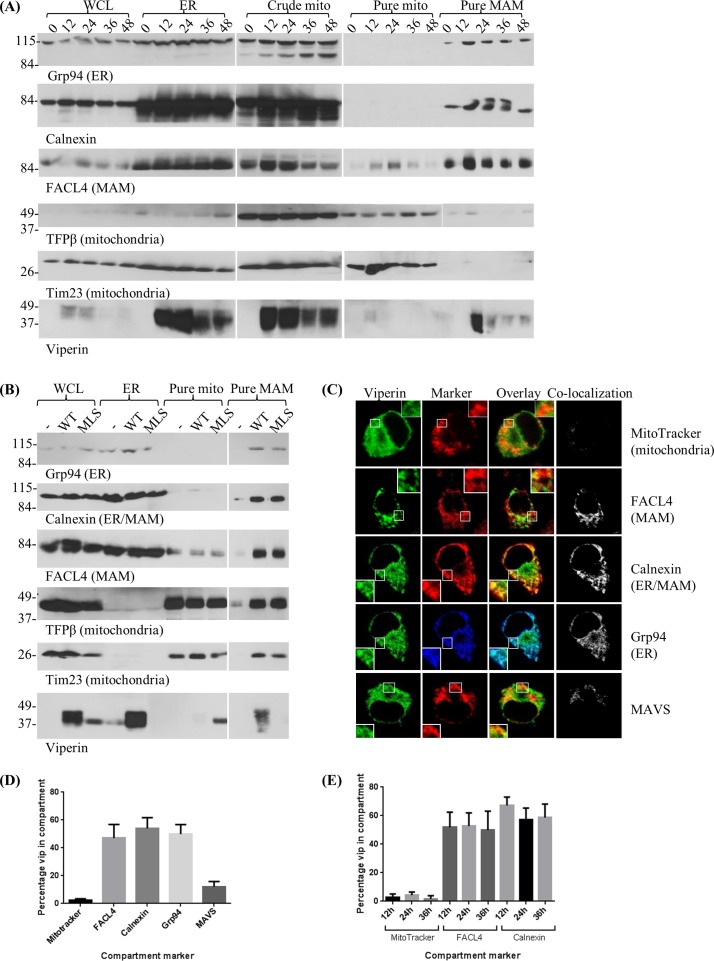
Localization of IFN-induced viperin and MAVS in MAM. **(A)** RAW 264.7 cells were untreated (0), or treated with universal Type I IFN (10^3^ units/ml) for 12, 24, 36, or 48 hours. Cells were harvested and subjected to Percoll fractionation. **(B)** 293T cells were either untransfected (-), transfected with pMX-IRES-Thy1.1-WT mouse viperin (WT) or pMX-IRES-Thy1.1 encoding mouse viperin with a mitochondrial localization sequence (MLS-viperin) then fractionated. **(C)** Mouse BMM were stimulated with IFN (10^3^ units/ml), stained for viperin and various other compartment markers and examined by confocal microscopy. The ‘co-localization’ column displays images generated by the ImageJ Co-localization plug-in; overlapping pixels are represented as white dots. **(D)** Co-localization was quantified using ImageJ software, presented as a percentage of total viperin that co-localizes with each compartment marker. Results are presented as ±SEM of least 10 different cells. **(E)** Mouse BMM were stimulated for varying lengths of time before being stained and viewed. Quantification of co-localization was performed as in panel D. Results are presented as ±SEM of least 10 different cells.

The localization of viperin to the endoplasmic reticulum (ER) is well established [32, 33], but potential localization to MAM has not been examined. We therefore used subcellular fractionation to examine the overall viperin distribution pattern. RAW 264.7 cells were stimulated with IFN and disrupted. The resulting membranes were then fractionated on a Percoll gradient to isolate the ER, mitochondria, and MAM. We were able to detect viperin in the ER and MAM fractions but not in the purified mitochondrial fraction. Localization to MAM was strong and consistent, and distribution did not change over the course of 48 hours (**Fig 1A**). This overall distribution was also observed in 293T cells transfected with either wild type (WT) viperin or viperin tagged with a mitochondrial localization sequence derived from vMIA (MLS-viperin) [10]. WT viperin was found in the MAM fraction, while MLS-viperin co-purified with the mitochondria (**Fig 1B**).

**Fig 2:**
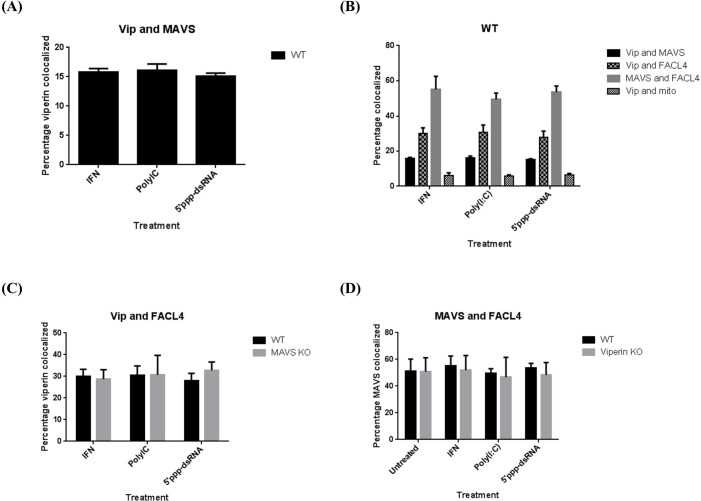
Viperin and MAVS do not influence each other’s intracellular distribution. **(A)** WT mouse BMM were stimulated with universal type I IFN or transfected with poly(I:C) or 5’ppp-dsRNA and co-localization of viperin with MAVS quantified after 8 hrs as in Fig 1. **(B)** WT mouse BMM were stimulated with universal type I IFN or transfected with poly(I:C) or 5’ppp-dsRNA for 8 hours and co-localization of various marker pairs quantified. **(C)** WT and MAVS KO mouse BMM were stimulated for 8 hours and co-localization of viperin with the MAM marker FACL4 was quantified. **(D)** WT and viperin KO mouse BMM were stimulated for 8 hours and co-localization of MAVS with FACL4 was quantified. All results are presented as ±SEM of least 10 different cells.

We also used confocal immunofluorescence microscopy to examine viperin and MAVS distribution in mouse bone marrow macrophages (BMM) stimulated with IFN, quantitating the percentage of the proteins that co-localized with each other and with markers for various intracellular compartments. Viperin co-localized with MAVS and the ER markers calnexin and Grp94, as well as the MAM marker FACL4, but not with mitochondria, detected using the specific dye MitoTracker (**Fig 1C and 1D**). This pattern did not change with time (**Fig 1E**). To determine whether the method of stimulation could affect localization patterns and whether viperin and MAVS could affect one another’s localization, WT, viperin knockout (KO), and MAVS KO BMM were stimulated with IFN or transfected with poly(I:C) or 5’ppp-dsRNA, which stimulate by interacting with MDA-5 and RIG-I, respectively [34–37], and the extent of co-localization was determined by confocal immunofluorescence microscopy. There was no significant difference in the localization patterns dependent on the method of stimulation, and the expression of viperin did not affect the distribution of MAVS or *vice versa* (**Fig 2A–2D**). This suggests that viperin and MAVS independently localize to the relevant compartments.

### Viperin interacts with MAVS

**Fig 3:**
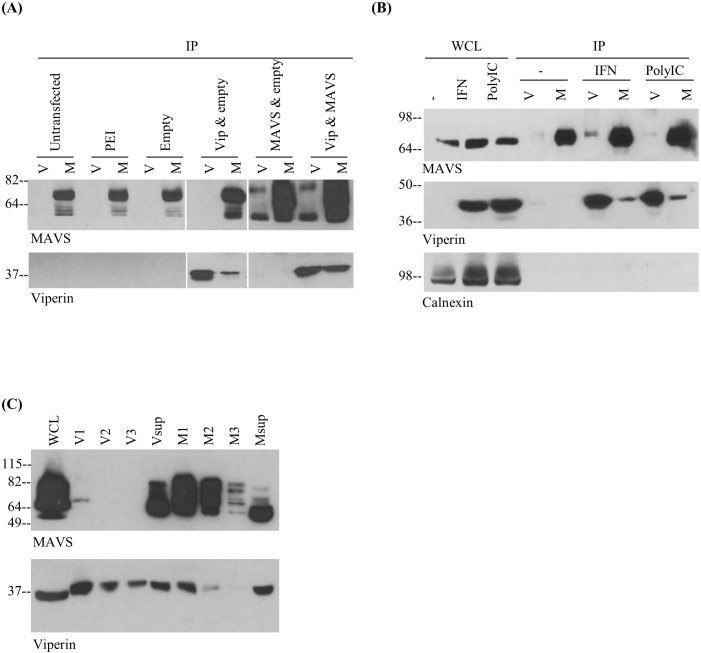
Viperin and MAVS interact. **(A)** 293T cells were untransfected (with only the transfection reagent present), or transfected with combinations of empty vector, pcDNA3.1-viperin, or pCMV-FLAG-MAVS. Cell lysates were subjected to immunoprecipitation (IP) with anti-viperin beads (V) or anti-MAVS beads (M) and blotted for viperin or MAVS. **(B)** RAW cells were stimulated with IFN (10^3^ units/ml) or transfected with poly(I:C) (1μg/ml) for 8h. Cell lysates were subjected to immunoprecipitation and blotted for viperin, MAVS, or calnexin as a negative control. **(C)** 293T cells were co-transfected with pcDNA3.1-viperin and pCMV-FLAG-MAVS and solubilized in 1% CHAPS in PBS. Equal aliquots were immunoprecipitated with anti-viperin beads or anti-MAVS beads. Beads were collected (V1 and M1 respectively), and the supernatants added to fresh beads for second (V2, M2) and third (V3, M3) immunoprecipitation. After immunoprecipitation, beads were collected (V2 and M2), and the process repeated. WCL is whole cell lysate without immunoprecipitation and Vsup and Msup designate the residual lysates after the third round of precipitation.

Given the involvement of both MAVS and viperin in defense against RNA viruses, as well as their co-localization in MAM, we considered the possibility that they might physically interact. Indeed, co-immunoprecipitations performed using detergent extracts of 293T cells co-transfected with viperin and MAVS demonstrated an interaction between the two (**Fig 3A**). To show that the association was not dependent on overexpression, co-immunoprecipitations were also performed using RAW 264.7 cells stimulated with IFN or transfected with poly(I:C), which activates cells via MDA-5 and the MAVS pathway. In both cases viperin was co-immunoprecipitated with an anti-MAVS antibody and MAVS with an anti-viperin antibody, indicating that they interact under physiological conditions (**Fig 3B**). Of note, some co-immunoprecipitation was observed even in extracts of unstimulated cells, likely a consequence of low basal levels of viperin present. To quantitate the extent of the interaction, a series of sequential immunoprecipitations were performed. The effective depletion of viperin from the extract left significant residual MAVS in the cell extract and *vice versa*, showing that only a fraction of the two components were co-associated (**Fig 3C**). Although this may to some extent reflect dissociation of MAVS and viperin upon detergent extraction, it does correlate well with co-localization quantifications described in **Figs 1 and 2**.

### Viperin residues involved in interaction with MAVS

**Fig 4:**
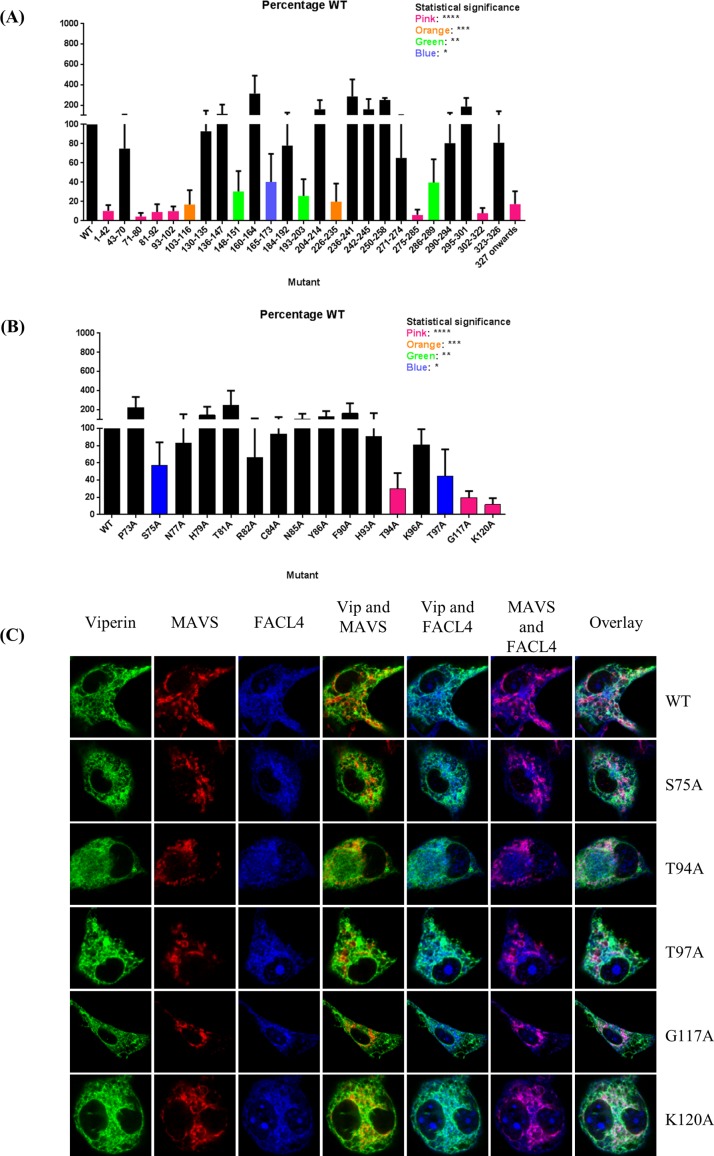
Mutational analysis of the viperin-MAVS interaction. **(A)** 293T cells were co-transfected with MAVS and the panel of viperin mutants made based on the unpublished viperin structure. Co-immunoprecipitations and western blots were performed as in Fig 3, and the interaction quantified as a ratio of [(viperin precipitated with anti-MAVS antibody) divided by (viperin precipitated with anti-viperin antibody)]. All values were normalized to the ratio obtained for wild type viperin in each experiment. Results are presented as ±SEM of least 3 independent experiments. **(B)** A series of point mutants of viperin were made and their interactions with MAVS similarly quantified. Results are presented as ±SEM of least 3 independent experiments. **(C)** Viperin knockout mouse BMM were transduced with constructs encoding viperin or the indicated mutants tethered to a tet-on promoter system. The cells were treated with doxycycline and examined by confocal immunofluorescence microscopy.

As a preliminary approach to determining the regions of viperin that interact with MAVS, we generated a series of deletion mutants corresponding to short segments of viperin, such as an α-helix or a loop, based on its as yet unpublished crystal structure (Y. Li, M. K. Fenwick, P. Cresswell, Y. Mordis, and S. E. Ealick, manuscript in preparation). The mutants were co-expressed with MAVS in 293T cells and immunoprecipitations were performed on detergent extracts of the cells followed by SDS-PAGE and quantitative western blotting. The data indicated that deletions at the N and C termini of viperin significantly reduced the viperin-MAVS interaction (**Fig 4A**). Western blots were checked to ensure that expression levels of the mutants were comparable to that of wild type viperin (**S1A Fig**).

Because the structure of the N terminal region of viperin was undefined in the crystal structure, the sequence was assessed using the ConSurf program [38] to predict the most likely exposed residues. Point mutations were made for 16 residues, converting them to alanines. As before, these mutants were co-expressed with MAVS in 293T cells and the level of interaction determined by quantitative western blotting. Five viperin residues appeared to be particularly important for the interaction: S75, T94, T97, G117, and K120 (**Fig 4B**). Similarly, Western blots were checked to ensure that expression levels of the mutants were comparable to that of wild type viperin (**S1B Fig**). Notably, when expressed in BMM using a doxycycline-dependent transduction system and analysed by confocal immunofluorescence microscopy, all of the mutants had a similar intracellular profile as WT viperin, consistent with the data in **Fig 2** showing that the viperin-MAVS interaction does not affect viperin distribution (**Fig 4C**).

### Viperin reduces IFNβ production upon cell stimulation

**Fig 5:**
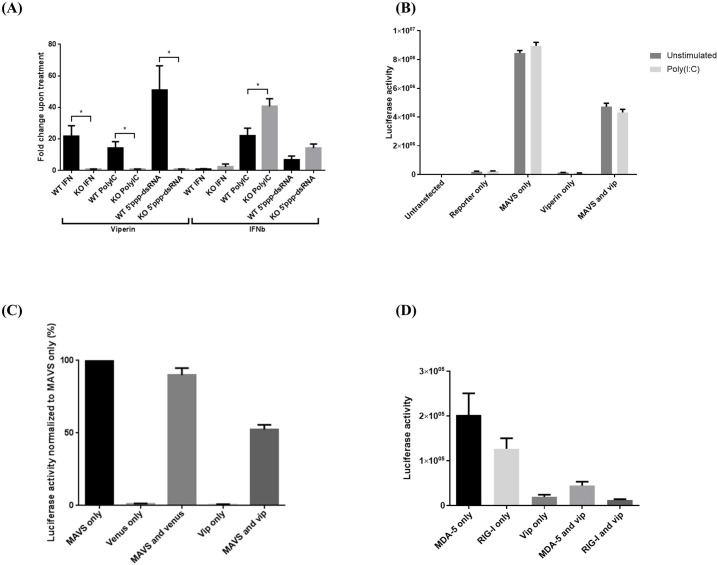
Viperin suppresses MAVS-dependent IFNβ induction. **(A)** WT and viperin KO BMM were treated with IFN at 10^3^ units/ml, or transfected with poly(I:C) at 1μg/ml or 5’ppp-dsRNA at 0.1μg/ml for 8 hours. qPCR analysis was performed to quantitate viperin and IFNβ mRNA expression. Results are presented as ±SEM of 4 independent experiments. **(B)** 293T cells were left untransfected or transfected with various combinations of the IFN reporter construct, MAVS, and viperin, and empty vector pcDNA3.1-myc-his where necessary to ensure equal amounts of DNA. Transfected cells were left untreated or transfected with poly(I:C) for 8 hours then subjected to luciferase activity assays. Results are presented as ±SD of quadruplicates. **(C)** 293T cells were transfected with 0.8μg of varying combinations of pCMV-FLAG-MAVS, pcDNA3.1-viperin, or, as a control, pcDNA3.1-Venus. Cells were lysed and lysates subjected to luciferase activity assays. Results are presented as ±SD of quadruplicates. **(D)** 293T cells were transfected with the IFN reporter construct and MDA-5 alone or together with viperin and subjected to luciferase activity assays. Results are presented as ±SD of quadruplicates.

To determine whether the viperin-MAVS interaction affects the functional consequences of MAVS-dependent signaling, BMM from WT and viperin KO mice were transfected with poly(I:C) or 5’ppp-dsRNA or, as a control, stimulated with universal type I IFN. IFNβ expression was monitored by quantitative PCR. Surprisingly, we found that viperin KO BMM expressed significantly higher levels of IFNβ than WT BMM upon poly(I:C) transfection. A similar trend was also seen upon IFN and 5’ppp-dsRNA stimulation, but it was much less pronounced than with poly(I:C) (**Fig 5A**). The focus was placed on these three ligands because IFNβ expression was very low with other modes of stimulation (data not shown).

To further examine the functional relationship between MAVS and viperin, we measured IFNβ expression in 293T cells expressing an IFNβ promoter (ISRE)-activated luciferase reporter construct. We found that poly(I:C) transfection alone was unable to elicit a strong response in these cells, as expected [39], and viperin expression alone also had little effect while overexpression of MAVS dramatically increased the signal in a manner unaffected by poly(I:C) transfection. However, consistent with the data obtained with WT and viperin knockout macrophages, co-expression of viperin reduced the signal compared to MAVS alone (**Fig 5B**). Co-expression with MAVS of the GFP derivative Venus, as a control, marginally reduced IFNβ expression, possibly because of promoter competition. However, the reduction caused by viperin co-expression was much more significant (**Fig 5C**). Transfecting MDA-5 or RIG-I alone increased the signal to a much lesser extent than MAVS, but again the signal was reduced when viperin was co-transfected (**Fig 5D**).

### Increased IRF-3 phosphorylation in viperin knockout macrophages

**Fig 6:**
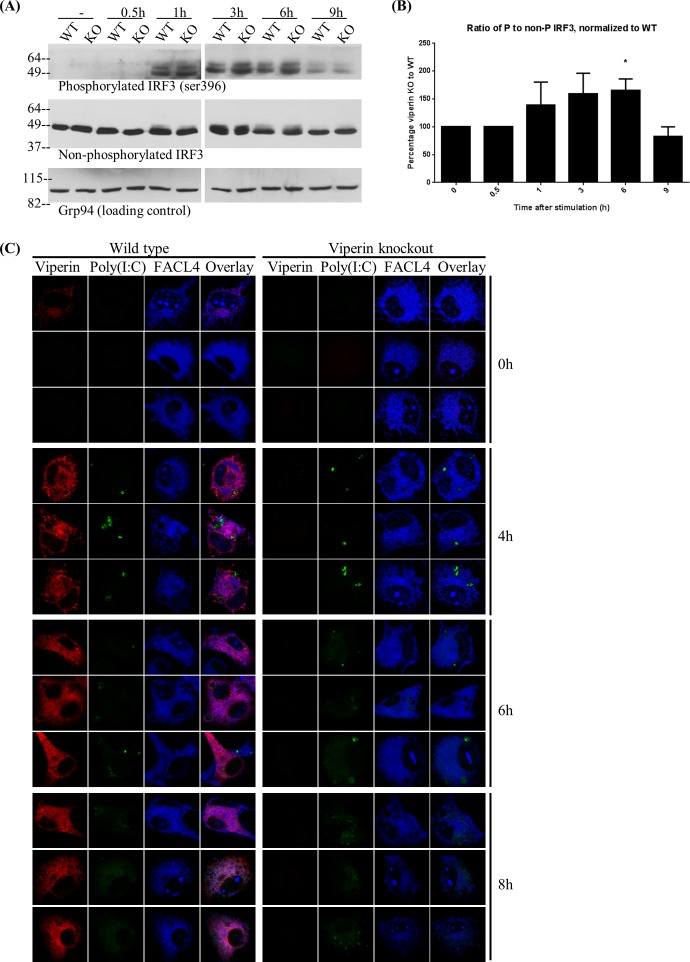
Viperin suppresses MAVS-dependent signaling. **(A)** WT and viperin KO BMM were transfected with poly(I:C) for varying lengths of time, lysed and subjected to SDS-PAGE and western blotting using antibodies to phosphorylated or unphosphorylated IRF3 or Grp94 as a control. **(B)** Quantification of the data in (A), presented as ±SEM of three independent experiments. **(C)** Confocal immunofluorescence microscopy on WT or viperin KO BMM transfected with fluorescein-tagged poly(I:C) at 0.1μg/ml for varying lengths of time. Representative cells are shown. Note that in WT BMM viperin can be detected in some cells at 4h and 6h but expression is much more widespread at later time points.

Reduction in IFNβ transcripts in the absence of viperin could because of suppressed signaling or post-transcriptional effects of viperin. We therefore asked directly whether signaling was affected by measuring phosphorylation of IRF3, which in the MAVS signaling pathway precedes the transcription of IFNβ and other pro-inflammatory genes. In viperin KO BMM, there is increased IRF-3 phosphorylation, particularly at the 6h time point (**Fig 6A and 6B**), suggesting that viperin is indeed a negative regulator of MAVS-dependent signaling. To ensure that viperin KO macrophages have normal accumulation of transfected poly(I:C), we used fluorescent poly(I:C). There was no significant difference between the levels accumulated by WT and viperin KO BMM, when analysed via confocal immunofluorescence microscopy (**Fig 6C**).

### The viperin-MAVS interaction restricts viperin-mediated IFNβ inhibition

**Fig 7:**
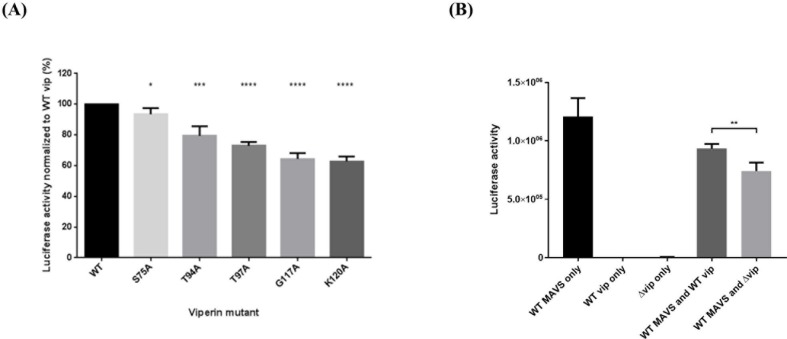
The ability of viperin mutants to bind MAVS inversely correlates with signal suppression. **(A)** 293T cells were co-transfected with the IFNβ luciferase reporter construct, pCMV-FLAG-MAVS, and pcDNA3.1-viperin or its mutants then subjected to luciferase activity assays. Results are presented as ±SEM of three independent experiments, each with quadruplicates. **(B)** Soluble viperin, lacking the N terminal α-helix (Δvip), was analysed using the luciferase reporter system as described above. Results are presented as ±SD of quadruplicates.

We next asked whether the reduction in MAVS-dependent signaling mediated by viperin depended upon the viperin/MAVS interaction. To determine this we again used the 293T luciferase reporter system. MAVS was co-expressed with the viperin point mutants that significantly reduced the viperin-MAVS interaction shown in **Fig 4B**, namely S75A, T94A, T97A, G117A, and K120A. Surprisingly, suppression of IFNβ production correlated inversely with the apparent strength of the viperin-MAVS interaction. For S75A, which was more similar to WT viperin in its interaction with MAVS, the reduction in IFNβ transcripts was barely statistically different from WT viperin. However, for the mutants that had more pronounced effects on the interaction, T94A, T97A, G117A, and K120A, IFNβ production was much reduced, indicating that the mutants are better inhibitors than WT viperin (**Fig 7A**). This suggests that viperin-mediated inhibition of MAVS-mediated signaling may not dependent on a direct effect of viperin binding to MAVS. Potentially an indirect mechanism, involving sequestration of viperin via the MAVS interaction prevents viperin from acting in conjunction with other partners and pathways to inhibit IFNβ production. However, the majority of MAVS does not interact with viperin, based both on the fraction that co-immunoprecipitates as well as the small overlap in the intracellular distribution of the two components. This suggested that only the sequestration of viperin that is in the same subcellular region as MAVS, likely the MAM, is capable of interfering with signaling.

### IFNβ inhibition is independent of the viperin N-terminal α-helix

The N-terminal α-helix of viperin, extending from residues 1–42, is essential for its localization to membranes and lipid droplets [33, 40]. To further address the role of MAM localization of viperin in its ability to suppress MAVS-dependent signaling, we compared WT and soluble Δ1–42 viperin for their ability to inhibit IFNβ generation. If anything, soluble viperin was a slightly better inhibitor than WT viperin when co-transfected with MAVS (**Fig 7B**). This corresponds with interaction data shown in **Fig 4A**, and also indicates that, at least in the over-expression system used here, MAM localized viperin may not be the only form that can inhibit MAVS-dependent signals.

## Discussion

Viperin is membrane bound by virtue of its N-terminal amphipathic α-helix. It was previously shown to associate with the cytosolic face of the ER and with lipid droplets, and this manuscript extends those findings by showing that a fraction is also localized to MAM. The MAM compartment is known to be enriched in cholesterol and is involved in multiple processes, including lipid transport, calcium homeostasis, oxidative metabolism, apoptosis, autophagy, signalling, and viral infections [41, 42]. Viperin expression affects lipid metabolism and lipid raft formation [8, 9, 43] and its localization in the MAM may underlie some of these properties. When viperin is deliberately targeted to mitochondria by replacement of the N-terminal amphiphathic α-helix with a defined mitochondrial localization sequence, it interferes with fatty acid β-oxidation by interacting with the mitochondrial trifunctional protein [10]. The contiguity of MAM and mitochondria may facilitate a similar, but lesser, effect in the case of cells over-expressing normal viperin, which we have previously shown exhibit glycolytic acidification of the growth medium [8, 9]. This effect is massively exaggerated in HCMV infected cells by the HCMV-encoded protein vMIA, which binds viperin and delivers it to mitochondria, resulting in increased fatty acid biosynthesis and membrane accumulation used by the virus for envelope formation [10].

The data presented here show that a fraction of both IFN-induced viperin and, as previously shown [28, 29], MAVS, is present in MAM, and we also present evidence that the two proteins physically interact. We further identified five single amino acid substitution mutants of viperin that exhibit a reduced interaction. MAVS is the central cytosolic signalling adaptor for viral RNA recognition and the fact that viperin is an antiviral protein suggests a potential role for this interaction in the response to RNA viruses. It appears that viperin negatively regulates MAVS-dependent signaling, since there is increased IRF-3 phosphorylation in viperin KO BMM upon stimulation, and consistent with this we saw increased IFNβ expression in these cells compared to WT BMM, particularly in the MDA-5 pathway and stimulation by poly(I:C). The results with BMM are also strongly supported by a clear reduction in luciferase induction when viperin and MAVS are co-expressed in the 293T luciferase IFN reporter system. This seems in conflict with previously published studies, showing that viperin enhances TLR-7 and -9 signaling in plasmacytoid dendritic cells. This may be explained by the fact that these experiments were performed in different cell types, which can respond differently upon activation of the same pathway [44] and, in addition, the different anti-viral signaling pathways used.

The mechanism by which viperin negatively regulates MAVS-dependent MDA-5 mediated signalling is unclear. A straightforward explanation would have been that the interaction between viperin and MAVS inhibits MAVS functions directly, for example by causing conformational changes in MAVS or competing for essential binding partners. In this case, however, one would expect that the interaction mutants would show a reduced capacity to inhibit IFNβ production. However, the converse was seen: the viperin-MAVS interaction appears to be required to restrain the inhibitory effect of viperin on IFNβ production, since viperin mutants with reduced interaction are better inhibitors of IFNβ production. It is possible that the mutant versions of viperin may have lost to varying degrees a functional capacity that is independent of their ability to bind to MAVS, and/or under basal conditions viperin could be inhibiting IFNβ production through the involvement of other as yet unknown factors and or even in a different pathway entirely. One possible explanation is that the interaction with MAVS sequesters viperin, preventing it from performing a critical inhibitory function. Although the molecular details of the inhibitory activity are unclear, at least in the over-expression system using 293T cells it does not depend on the N-terminal α-helix of viperin, suggesting that the effect of viperin on signalling is independent of membrane association.

We hypothesize that when a virus enters the cell, MAVS signaling is initiated very quickly, sequestering the low levels of viperin that are initially induced and allowing the cell to initiate a functional antiviral response. Later in the infection we suggest that increased viperin expression, which peaks at between 6 and 8 hours after stimulation, overwhelms the sequestration mediated by its interaction with MAVS. This allows it to inhibit the pro-inflammatory response and prevent immune-mediated pathology. The hypothesis that the population of viperin that mediates the inhibitory effect functions outside a MAVS-associated complex is supported by the observation that the majority of viperin does not interact with MAVS, and indeed is distributed differently within the cell.

The segregation of viperin into different subpopulations could also explain why a protein so widely thought to be involved in anti-viral defense is also a regulator. A key point may be that pathology in viral infections is often caused not directly by the virus itself, but by the host’s response to the infection, which can lead to cell death, tissue damage, and other systemic effects. Viperin is important for host survival in certain viral infections [4, 5, 7, 45], and the prevention of pathology caused by excessive inflammation could be a major contributing factor. A pro-inflammatory response is important at the beginning of the infection, but at later time points a regulatory system may be necessary to shut off the response. Viperin may play such a regulatory role specifically in the MAVS pathway, as opposed to the TLR-7 and -9 pathways.

We attempted to look for support for the proposed role of viperin in the inhibition of MAVS-mediated signaling by examining overall mRNA expression in RNAseq experiments performed on WT or viperin KO BMM, either unstimulated or stimulated with IFN (**S1 Table**). Multiple ISGs were identified, including IFIT3 and TRIM56, which are directly involved in MAVS-mediated or related pathways. Others, such as ISG15, USP18, and UBE2L6, are involved in ISGylation. Notably, USP18, a negative regulator of IFN signalling, is down-regulated in viperin KO BMM, suggesting that viperin may be required for its expression. Viperin may work together with or influence USP18 function, but further experiments are required to address this possibility. Nevertheless, our data show that viperin functions as a negative regulator of MAVS-mediated signalling, particularly in the MDA-5 pathway, where its induction is clearly downstream of MAVS activation. It interacts with MAVS, most likely in MAM, and this interaction is important in controlling its inhibitory activity, likely by some form of sequestration.

If viperin is a key player in MDA-5 and MAVS-mediated immune responses to RNA virus infections, it may provide a novel target for therapeutic interventions; although appropriate antiviral defence mechanisms are essential for viral clearance, excessive immune responses often account for the pathology seen in viral infections. Future studies placing viperin precisely in the signalling network may suggest approaches to tuning the immune response to either facilitate viral clearance or reduce immune pathology.

## Supporting information

S1 Fig Expression levels of viperin mutants are comparable to that of wild type viperin. **(A**) Western blots showing that deletion mutants are expressed at similar levels to that of wild type viperin. Each set of blots is from an individual experiment. All the blots shown here cover the entire range of deletion mutants, with some overlaps. **(B)** Western blots showing that point mutants are expressed at similar levels to that of wild type viperin. Each set of blots is from an individual experiment. All the blots shown here cover the entire range of point mutants, with some overlaps.
(TIF)

S1 Table Viperin affects the expression of multiple ISGs. A number of ISGs were expressed at significantly different levels in WT and viperin KO mouse BMM under basal conditions and upon IFN stimulation. Most of these were higher in WT than viperin KO cells in the absence of stimulation, suggesting that basal levels of viperin may enhance their expression. Descriptions were summarized from information provided in GeneCards (Borden EC, Sen GC, Uze G, Silverman RH, Ransohoff RM, Foster GR, et al. Interferons at age 50: past, current and future impact on biomedicine. Nature reviews Drug discovery. 2007;6(12):975–90).
(XLSX)
