# Supplementary figures and images for: Viperin interaction with mitochondrial antiviral signaling protein (MAVS) limits viperin-mediated inhibition of the interferon response in macrophages
Source: PLoS One. 2017 Feb 16;12(2):e0172236. doi: 10.1371/journal.pone.0172236 (PMC5313200; doi:10.1371/journal.pone.0172236)

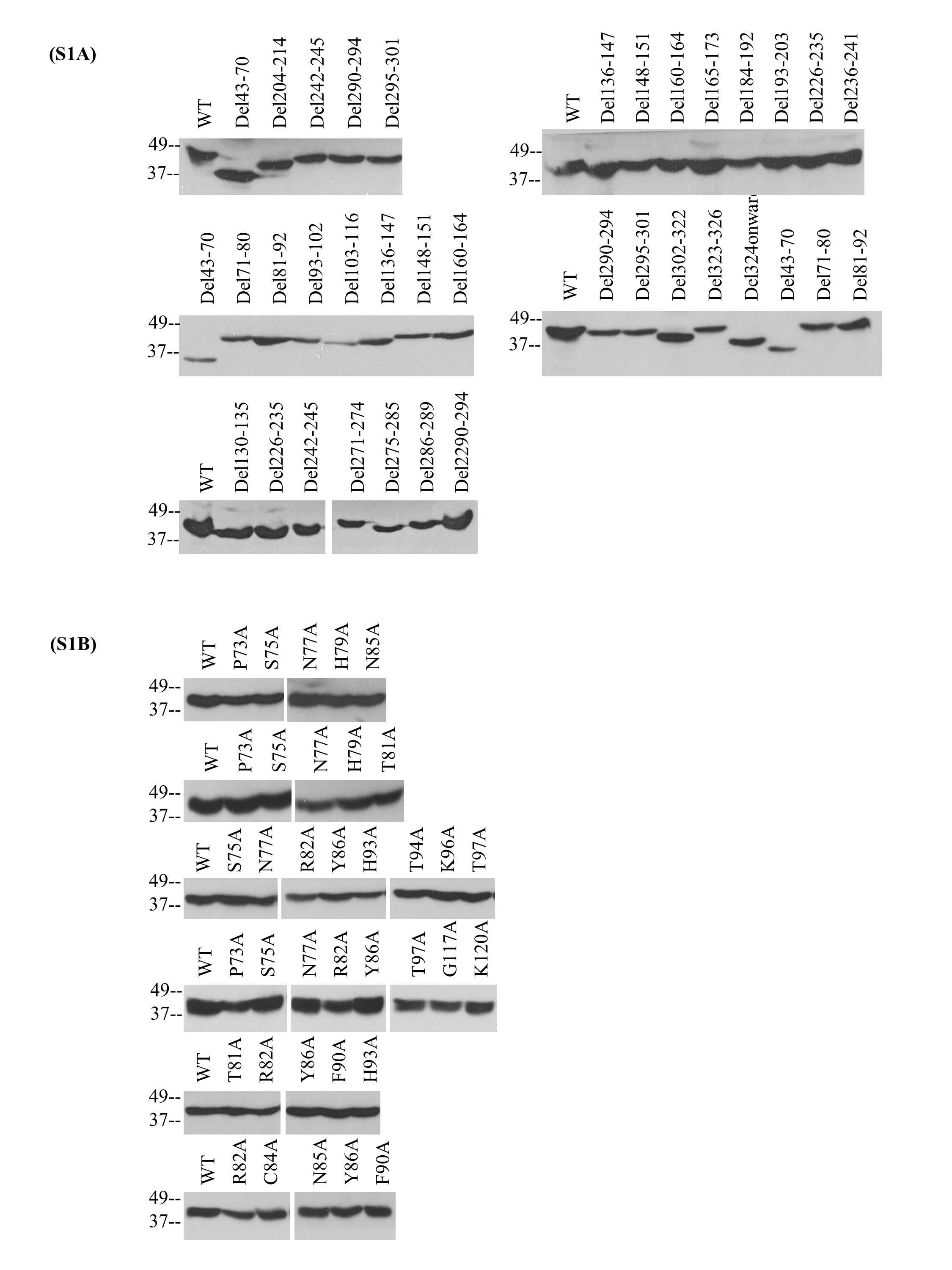

Supplement: S1 Fig — (A) Western blots showing that deletion mutants are expressed at similar levels to that of wild type viperin. Each set of blots is from an individual experiment. All the blots shown here cover the entire range of deletion mutants, with some overlaps. (B) Western blots showing that point mutants are expressed at similar levels to that of wild type viperin. Each set of blots is from an individual experiment. All the blots shown here cover the entire range of point mutants, with some overlaps. (TIF) [file pone.0172236.s001.TIF]
